# Karyotype and putative chromosomal inversion suggested by integration of cytogenetic and molecular data of the fungus-farming ant *Mycetomoellerius iheringi* Emery, 1888

**DOI:** 10.3897/CompCytogen.v14i2.49846

**Published:** 2020-05-07

**Authors:** Ricardo Micolino, Maykon Passos Cristiano, Danon Clemes Cardoso

**Affiliations:** 1 Programa de Pós-Graduação em Genética, Departamento de Genética, Universidade Federal do Paraná (UFPR), Centro Politécnico, Jardim das Américas, 81531-990,Curitiba, PR, Brazil Universidade Federal do Paraná Curitiba Brazil; 2 Departamento de Biodiversidade, Evolução e Meio Ambiente, Universidade Federal de Ouro Preto (UFOP), Ouro Preto, MG, Brazil Universidade Federal de Ouro Preto Ouro Preto Brazil

**Keywords:** chromosomal evolution, FISH, fungus growing, karyomorphometry, TTAGG, *
Trachymyrmex
*

## Abstract

Comparative cytogenetic analyses are being increasingly used to collect information on species evolution, for example, diversification of closely related lineages and identification of morphologically indistinguishable species or lineages. Here, we have described the karyotype of the fungus-farming ant *Mycetomoellerius iheringi* Emery, 1888 and investigated its evolutionary relationships on the basis of molecular and cytogenetic data. The *M. iheringi* karyotype consists of 2n = 20 chromosomes (2K = 18M + 2SM). We also demonstrated that this species has the classical insect TTAGG telomere organization. Phylogenetic reconstruction showed that *M. iheringi* is phylogenetically closer to *M. cirratus* Mayhé-Nunes & Brandão, 2005 and *M. kempfi* Fowler, 1982. We compared *M. iheringi* with other congeneric species such as *M. holmgreni* Wheeler, 1925 and inferred that *M. iheringi* probably underwent a major pericentric inversion in one of its largest chromosomes, making it submetacentric. We discussed our results in the light of the phylogenetic relationships and chromosomal evolution.

## Introduction

Fungus-farming ants (Formicidae: Myrmicinae: Attini) are exclusive to the New World and occur mainly in the Neotropical region, with some species found in the Nearctic region (Weber 1966; Rabeling et al. 2007). The most recently diverged species include the well-known leafcutter ants (genera *Atta* Fabricius, 1804 and *Acromyrmex* Mayr, 1865) as well as the genera *Xerolitor* Sosa-Calvo et al., 2018, *Sericomyrmex* Mayr, 1865 and *Trachymyrmex* Forel, 1893. Previous phylogenetic analyses have shown that the genus *Trachymyrmex* is paraphyletic (e.g., Schultz and Brady 2008; Sosa-Calvo et al. 2018; Micolino et al. 2019a). However, this taxonomic complication was recently resolved by multilocus phylogenetic analyses with a comprehensive number of species (Solomon et al. 2019). Thus, a new systematic arrangement of three clades was proposed as follows: *Mycetomoellerius*Solomon et al. 2019 (former *Iheringi* group), *Paratrachymyrmex* Solomon et al., 2019 (former *Intermedius* group), and *Trachymyrmex* (based on the type species *Trachymyrmex septentrionalis* McCook, 1881). Nevertheless, *Trachymyrmexsensu stricto*, largely containing North American species, is still most prominently studied (e.g., Rabeling et al. 2007; Seal et al. 2015; Sánchez-Peña et al. 2017).

Cytogenetics encompasses the study of chromosomes that may have direct implications on species evolution, such as the identification of cryptic species and diversification of closely related lineages (White 1978; King 1993). In general, ants exhibit one of the largest chromosomal variability among organisms (reviewed by Lorite and Palomeque 2010), leading to the hypothesis that chromosomal rearrangements, i.e., Robertsonian fissions and fusions (known major rearrangements that can change the chromosomal number within lineages), actively contributed to the diversification of ants (Imai et al. 1988, 2001; Cardoso et al. 2018a). Despite the large number of species in the three genera formerly included into “*Trachymyrmex*” (about 60 species, see above), there is limited cytogenetic information on this ant group. To date, only seven species have been karyotyped, three of which have not been identified to the species level (see Table 1). On the basis of the available data, the described chromosomal numbers appear to be stable within the three genera, ranging from 2n = 12 to 2n = 22 and predominantly comprising metacentric chromosomes (reviewed by Cardoso et al. 2018a).

**Table 1.** Former “*Trachymyrmex*” species with their described karyotypes. 2n: diploid chromosome number; (n): haploid chromosome number; 2K: karyotype formula; Locality: sampling site; M: metacentric chromosomes; SM: submetacentric chromosomes.

Species	2n (n)	2K	Locality	References
Mycetomoellerius fuscus*	18 (9)	16M + 2SM	Minas Gerais State, Brazil	Barros et al. (2013a)
*Mycetomoellerius holmgreni*	20 (10)	20M	Minas Gerais State, Brazil	Barros et al. (2018)
*Mycetomoellerius iheringi*	20 (10)	18M + 2SM	Santa Catarina State, Brazil	Present study
*Mycetomoellerius relictus*	20 (10)	20M	Minas Gerais State, Brazil	Barros et al. (2013b)
*Trachymyrmex septentrionalis*	20 (10)	20M	Barro Colorado Island, Panama	Murakami et al. (1998)
“*Trachymyrmex*” sp. 1	12 (6)	12M	Barro Colorado Island, Panama	Murakami et al. (1998)
“*Trachymyrmex*” sp. 2	18 (9)	18M	Barro Colorado Island, Panama	Murakami et al. (1998)
“*Trachymyrmex*” sp. 3	22 (11)	18M + 4SM	Minas Gerais State, Brazil	Barros et al. (2013b)

* current junior synonym of *M. urichii*.

*Mycetomoellerius iheringi* Emery, 1888, the type species of the genus, is a species endemic to South America, and it occurs mainly in the southern regions. The exclusive characteristic of *M. iheringi* is the finely striated discal area of the mandibles, which sets it apart from the congeneric species *Mycetomoellerius kempfi* Fowler, 1982 (Mayhé-Nunes and Brandão 2005). A feature of *M. iheringi* biology that facilitates field identification is the subterranean nest in the sand with a slim opening (Mayhé-Nunes and Brandão 2005). Some groups have been identified by morphological similarities within the former “*Trachymyrmex*”, including the *Iheringi* group that also includes *Mycetomoellerius holmgreni* Wheeler, 1925 whose karyotype has been already described (Mayhé-Nunes and Brandão 2005; Barros et al. 2018). This fact allows cytogenetic comparisons with *M. iheringi*. However, the phylogenetic position of *M. iheringi* has not yet been described; only the relationship between its fungal cultivars has been reported (see Solomon et al. 2019).

Here, we have described the *M. iheringi* karyotype on the basis of karyomorphometric analysis and fluorescence *in situ* hybridization (FISH) with a telomeric probe. In addition, we identified the phylogenetic position of *M. iheringi* and examined its relationship with other species of the genus. We have discussed our results in the light of chromosomal evolution among fungus-farming ants.

## Material and methods

### Colony sampling

Colonies of *M. iheringi* were collected from the Restinga environment of the Brazilian Atlantic coast at Joaquina Beach, Florianópolis, Santa Catarina State, Brazil (27°37'44"S; 48°26'52"W). A total of five distantly spaced colonies were sampled. Such colonies were maintained *in vivo* at the Laboratório de Genética Evolutiva e de Populações, Universidade Federal de Ouro Preto, Brazil, according to the protocol established by Cardoso et al. (2011).

### Chromosome preparation and FISH mapping

Metaphase chromosomes from the brain ganglia of pre-pupal larvae were obtained using the method of Imai et al. (1988). The ganglia were dissected under a stereomicroscope and incubated in hypotonic solution containing 1% sodium citrate and 0.005% colchicine for 60 min, and consecutively dissociated and fixed on stereoscopic microscope slides in acetic acid: ethanol: distilled water (3:3:4) and acetic acid: ethanol (1:1). Subsequently, the metaphase chromosomes were examined under a phase-contrast microscope and stained with 4% Giemsa stain dissolved in Sorensen’s buffer, pH 6.8, to determine the chromosome number and morphology. We classified the chromosomes according to the nomenclature proposed by Levan et al. (1964), which is based on the ratio of the chromosomal arms (*r*), given by centromere position. The chromosomes were classified into metacentric (*r* = 1.0–1.7), submetacentric (*r* = 1.7–3.0), subtelocentric (*r* = 3.0–7.0), and acrocentric (*r* > 7.0) categories, as modified by Crozier (1970). The metaphase chromosomes were measured using IMAGE-PRO PLUS software (Media Cybernetics, LP, USA), and the values were calibrated by the scale bar and transferred to EXCEL (Microsoft, Redmond, WA, USA). In addition, the degree of variation and karyotype measurement were validated using statistical tests, according to Cristiano et al. (2017).

FISH experiments were performed as previously described by Kubat et al. (2008), with detailed modifications for ants by Micolino et al. (2019a). For the hybridizations, we used the TTAGG_(6)_ telomeric motif, which has fine conservation in most insects and the advantage of being able to detect chromosomal rearrangements such as telomere-related inversions and fusions. The TTAGG_(6)_ probe was directly labeled with Cy3 at the 5' terminal during synthesis (Sigma, St. Louis, MO, USA). The summarized technique involves several saline washes, alcohol dehydration, and formamide denaturation, until hybridization with the probe. For visualization, the metaphase chromosomes were stained with 4',6-diamidino-2-phenylindole (DAPI Fluoroshield, Sigma-Aldrich) in an antifade solution. The metaphase chromosomes were analyzed under an OLYMPUS BX53 epifluorescence microscope with OLYMPUS CELLSENS IMAGING software (Olympus American, Inc., Center Valley, PA, USA), using WU (330–385 nm) and WG (510–550 nm) filters for DAPI and rhodamine, respectively. About 10–20 metaphases were analyzed in both cytogenetic analyses, and the images were edited with ADOBE PHOTOSHOP CC software.

### DNA extraction, sequencing, and phylogenetic analysis

We extracted the DNA from *M. iheringi* ant workers, according to the standard CTAB/chloroform technique (Sambrook and Russell 2001). We sequenced the fragments of four nuclear genes, *elongation factor 1-alpha-F1* (EF1α-F1), *elongation factor 1-alpha-F2* (EF1α-F2), *wingless* (Wg), and *long-wavelength rhodopsin* (LW Rh), and one mitochondrial gene, *cytochrome c oxidase I* (COI) (GenBank accession numbers: MT174160–MT174169). The primers used to generate the sequence data are listed in Table 2. Polymerase chain reaction was performed using a final volume of 25 μL, according to the manufacturer’s instructions (Promega, Madison, WI, USA). The amplification conditions and sequencing were based on the methodology outlined in previous studies (see Schultz and Brady 2008, Cardoso et al. 2015a, b, Ward et al. 2015).

The gene fragments were aligned and concatenated using MEGA7 software (Kumar et al. 2016) and incorporated into the dataset of Solomon et al. (2019). The phylogeny was inferred using the maximum likelihood criterion in RAxML (Stamatakis 2014) by using the simultaneous best-tree search and rapid bootstrapping analysis (1000 replicates) with the GTR + G model of evolution. The generated tree and branch labels were visualized using FIGTREE software (Rambaut 2009).

**Table 2.** Primers used for sequencing four nuclear (*EF1α-F1*, *EF1α-F2*, *Wg* and *LW Rh*) and one mitochondrial (*COI*) gene fragments in the fungus-farming ant *Mycetomoellerius iheringi*.

Gene region	Primer	Sequence 5' to 3'	Source
*EF1α-F1*	1424F	GCGCCKGCGGCTCTCACCACCGAGG	Brady et al. (2006)
	1829R	GGAAGGCCTCGACGCACATMGG	Brady et al. (2006)
*EF1α-F2*	557F	GAACGTGAACGTGGTATYACSAT	Brady et al. (2006)
	1118R	TTACCTGAAGGGGAAGACGRAG	Brady et al. (2006)
*LW Rh*	LR143F	GACAAAGTKCCACCRGARATGCT	Ward and Downie (2005)
	LR639ER	YTTACCGRTTCCATCCRAACA	Ward and Downie (2005)
*Wg*	wg578F	TGCACNGTGAARACYTGCTGGATGCG	Ward and Downie (2005)
	wg1032R	ACYTCGCAGCACCARTGGAA	Abouheif and Wray (2002)
*COI*	LCO1490	GGTCAACAAATCATAAAGATATTGG	Folmer et al. (1994)
	HCO2198	TAAACTTCAGGGTGACCAAAAAATCA	Folmer et al. (1994)

## Results

### Cytogenetic data

**Figure 1.**
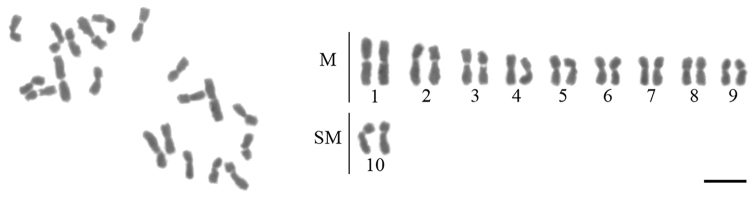
Mitotic metaphase of *Mycetomoellerius iheringi* with 2n = 20 chromosomes and its karyotypic morphology. M: metacentric chromosomes; SM: submetacentric chromosomes. Scale bar: 5 μm.

**Figure 2.**
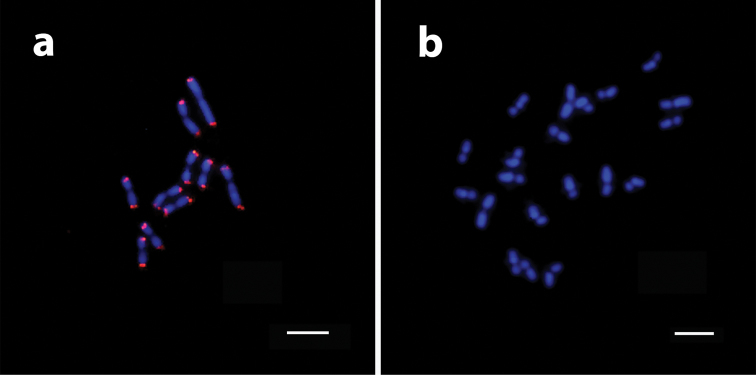
DAPI-stained *Mycetomoellerius iheringi* chromosomal metaphases **a**FISH mapping of the TTAGG_(6)_ telomeric motif on haploid metaphase **b** chromosomes uniformly stained with DAPI fluorochrome, except for the centromeric region. Scale bar: 5 μm.

The karyotype of *M. iheringi* has 2n = 20 chromosomes (Fig. 1). Our karyomorphometric analysis revealed that this karyotype consists of nine metacentric pairs and one submetacentric pair; the karyotype formula is 2K = 18M + 2SM, and the fundamental number is FN = 40. The total average length of all chromosomes (i.e., of the diploid karyotype) was estimated to be 82.51 ± 0.52 μm. The average chromosome length ranged from 5.77 ± 0.91 μm to 3.37 ± 0.4 μm (Table 3). The telomere distribution of the TTAGG_(6)_ motif was displayed at both ends of all *M. iheringi* chromosomes (Fig. 2a). No signals for interstitial telomeric sites (ITS) were detected using this probe. Moreover, DAPI staining revealed that both arms of all chromosomes were completely labeled, i.e., mostly A-T rich, whereas the centromeric region showed no labeling for this fluorochrome (Fig. 2b).

**Table 3.** Karyomorphometric analysis of the chromosomes of *Mycetomoellerius iheringi*. TL: total length; L: long arm length; S: short arm length; RL: relative length; *r*: arm ratio (= L/S); ∑: total average length of all chromosomes or Karyotype lenght (KL).

**Chromosome**	**TL**	**L**	**S**	**RL**	***r***	**Classification**
1	5.77±0.91	3.03±0.48	2.74±0.43	6.97±0.34	1.1±0.05	Metacentric
2	5.46±0.75	2.86±0.46	2.6±0.32	6.61±0.24	1.1±0.08	Metacentric
3	5.09±0.66	3.02±0.41	2.08±0.27	6.17±0.29	1.46±0.09	Metacentric
4	4.71±0.53	2.67±0.29	2.04±0.28	5.72±0.34	1.32±0.12	Metacentric
5	4.38±0.49	2.38±0.29	1.99±0.29	5.31±0.2	1.21±0.18	Metacentric
6	4.2±0.46	2.3±0.23	1.91±0.27	5.1±0.15	1.22±0.14	Metacentric
7	4.07±0.46	2.24±0.2	1.83±0.33	4.94±0.16	1.26±0.21	Metacentric
8	4.01±0.44	2.3±0.26	1.72±0.26	4.87±0.16	1.32±0.19	Metacentric
9	3.89±0.43	2.19±0.3	1.7±0.18	4.72±0.11	1.31±0.14	Metacentric
10	3.83±0.45	2.16±0.3	1.67±0.17	4.65±0.06	1.3±0.11	Metacentric
11	3.78±0.43	2.15±0.28	1.63±0.2	4.59±0.1	1.32±0.15	Metacentric
12	3.73±0.41	2.07±0.3	1.66±0.15	4.53±0.15	1.25±0.15	Metacentric
13	3.7±0.39	2.03±0.26	1.67±0.19	4.5±0.14	1.22±0.14	Metacentric
14	3.66±0.4	2.08±0.24	1.58±0.2	4.44±0.13	1.33±0.14	Metacentric
15	3.58±0.35	2.01±0.28	1.57±0.13	4.35±0.13	1.29±0.17	Metacentric
16	3.54±0.38	2.01±0.26	1.54±0.17	4.3±0.12	1.32±0.16	Metacentric
17	3.51±0.4	2.04±0.19	1.47±0.25	4.26±0.13	1.41±0.16	Metacentric
18	3.37±0.4	1.94±0.29	1.43±0.12	4.09±0.11	1.36±0.13	Metacentric
19	4.29±1.1	2.74±0.68	1.56±0.42	5.15±0.72	1.77±0.06	Submetacentric
20	3.94±0.59	2.51±0.37	1.43±0.22	4.76±0.25	1.76±0.03	Submetacentric
∑	**82.51±0.52**					

### Molecular data

**Figure 3.**
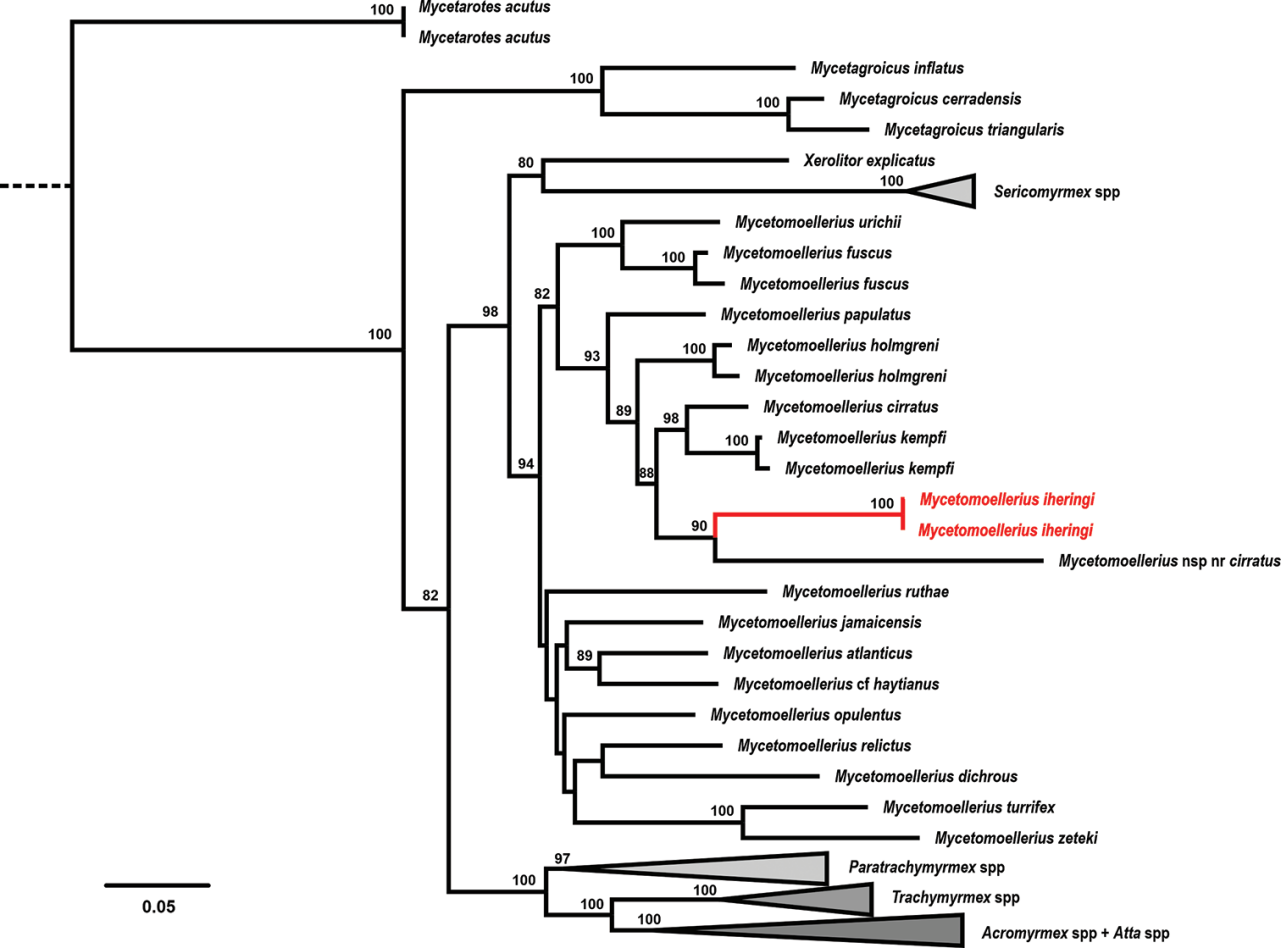
Maximum-likelihood phylogeny of “higher” fungus-farming ants generated in RAxML. *Mycetomoellerius iheringi* is indicated in red. Node numbers represent the bootstrapping values after 1000 replications; values < 80 are not shown. Scale bar indicates nucleotide substitutions per site.

The maximum likelihood phylogeny showed *M. iheringi* as the sister species of a lineage defined as Mycetomoellerius n. sp. nr cirratus (see Solomon et al. 2019) (bootstrap value, PB = 90). The clade composed of *M. cirratus* Mayhé-Nunes & Brandão, 2005 + *M. kempfi* (PB = 98) forms the sister group of *M. iheringi* + M. n. sp. nr cirratus (PB = 88). The species *M. holmgreni* previously diverged from the aforementioned clades (PB = 89), and *M. papulatus* Santschi, 1922 was estimated to be the most basal of the “*Iheringi* group” (PB = 93) (Fig. 3).

## Discussion

Here, we have provided the karyotypic description of the fungus-farming ant *Mycetomoellerius iheringi*, which has 2n = 20 chromosomes; we presented its phylogenetic position in the clade of the “*Iheringi* group”. Considering the cytogenetic data available from fungus-farming ants, we observed a numerical constancy among the karyotypes of the lineages that diverged most recently (i.e., leafcutter ants of the genera *Atta* and *Acromyrmex*), suggesting this karyotypic characteristic is shared by the relatively recent lineages. *Trachymyrmex septentrionalis*, a sister clade of leafcutter ants, has 2n = 20 metacentric chromosomes, equal to those of two *Mycetomoellerius* species, *M. holmgreni* and *M. relictus* Borgmeier, 1934 (see Table 1). All *Atta* species karyotyped to date have 2n = 22 chromosomes, and most *Acromyrmex* species have 2n = 38 (reviewed by Cardoso et al. 2018a). In other Hymenoptera species, such as stingless bees of the tribe Meliponini Lepeletier, 1836, this scenario can also be seen in the genera with a conserved chromosome number (Travenzoli et al. 2019).

In the new taxonomic status, *Mycetomoellerius* is composed of about 30 described species (Solomon et al. 2019), but only four have known karyotypes and, interestingly, a prevalence of metacentric chromosomes (see Table 1). The species *M. iheringi* and *M. holmgreni* are closely related morphologically (Mayhé-Nunes and Brandão 2005), and, as we have shown, *M. holmgreni* diverged previously from *M. iheringi*. Moreover, both species co-occur in southern Brazilian sand-dune habitats (Cardoso and Schoereder 2014). Importantly, the karyotypes of these two species are similar: they have analogous karyotype measurements and DAPI-staining pattern as well the chromosomal number 2n = 20, differing by only one pair of submetacentric chromosomes (Barros et al. 2018; Cardoso et al. 2018b). A likely, and the most parsimonious, scenario for explaining such cytogenetic differences would involve at least one major chromosomal rearrangement. Therefore, we suggest a pericentric inversion occurred in one of the larger *M. iheringi* chromosomes, resulting in the current karyotype morphology. Such chromosomal rearrangement could have occurred in any lineage of the clades underlying *M. holmgreni*; however, such lineages should be karyotyped to verify this hypothesis. The base chromosome number, defined as the haploid number present in the initial lineage of a monophyletic clade, may be directly related to the chromosomal variability within that clade (Guerra 2008). Thus, the assumption of this major inversion is attributable to the fact that *M. holmgreni* has a karyotype formed by only metacentric chromosomes, which becomes a putative ancestral characteristic of the underlying lineages, such as *M. iheringi*.

The application of classical and molecular cytogenetic techniques, such as chromosomal banding and FISH mapping, has increasingly contributed to comparative evolutionary studies. Because of new ant cytogenetic data, valuable information is being collected and correlated to their evolution and exceptional chromosomal diversity. For instance, fusion and fission rearrangements have been proposed to play a crucial role in the diversification of the fungus-farming ants of the genus *Mycetophylax* Emery, 1913 (Cardoso et al. 2014; Micolino et al. 2019b). Indeed, chromosomal changes may be directly related to the speciation process for a range of taxa (Rieseberg 2001; Faria and Navarro 2010). In particular, inversions are abundant in natural populations and can have several evolutionary implications, such as adaptation and divergence of lineages (Ayala and Coluzzi 2005; Wellenreuther and Bernatchez 2018). Inversion polymorphisms may contribute to speciation by reducing recombination and consequently protecting genomic regions from introgression (Hoffmann and Rieseberg 2008). Moreover, a model has predicted that closely related lineages that co-occur in a region could readily differ by one or more inversions because such lineages would persist longer in the face of gene flow than in the absence of these inversions (Noor et al. 2001). Our data support such a model, mainly because the species *M. iheringi* and *M. holmgreni* live sympatrically and are phylogenetically close.

The rich karyotypic diversity of ants deserves special attention. Inversion polymorphisms, for example, have been reported in many ant species. For example, intrapopulational polymorphism has been detected in the *Iridomyrmex gracilis* Lowne, 1865 complex. Such populations with the same chromosome number but distinct karyotype structures have led authors to propose that a pericentric inversion occurred in a metacentric chromosome, making it acrocentric (n = 6M + 1SM + 1A to n = 5M + 1SM + 2A) (Crozier 1968). The chromosome number and morphology of *Pachycondyla* Smith, 1858 are variable; their karyotypes show a predominance of submetacentric and acrocentric chromosomes, which allows the interpretation that fission and pericentric inversions (where metacentric chromosomes turn acrocentric or vice versa) would be the most frequent chromosomal rearrangements in the evolution of this genus and even contribute to the speciation processes (Mariano et al. 2012). The intraspecific chromosomal variability in social organization (monogyny vs. polygyny) found in the fire ant *Solenopsis invicta* Buren, 1972 can also be explained by at least one large inversion, which would account for a lack of recombination over more than half of the two heteromorphic “social chromosomes” (Wang et al. 2013).

Another interesting finding was reported in *Mycetomoellerius fuscus* Emery, 1894 (current junior synonym of *M. urichii* Forel, 1893, see Micolino et al. 2019a for discussion), a species with a geographic distribution similar to *M. iheringi* and *M. holmgreni* and found largely in southern South America (Brandão and Mayhé-Nunes 2007). They are phylogenetically closer than previously expected (Micolino et al. 2019a; Solomon et al. 2019). *Mycetomoellerius fuscus* has a chromosomal morphology of eight metacentric pairs and a submetacentric pair (2n = 18) (Barros et al. 2013a). As the submetacentric pair is the biggest chromosome of the karyotype, there could have been a Robertsonian fusion rearrangement, followed by a pericentric inversion, making it submetacentric. The other few species of “*Trachymyrmex*” with the described karyotype (see Table 1) do not allow us to picture a full scenario for the karyoevolution of the genera. Further, unidentified specimens vary relatively widely from 2n = 12 to 2n = 22. The karyotype 2n = 12 presented by Murakami et al. (1998) is quite intriguing, as this unidentified specimen could be a key piece to understanding the chromosomal evolution of the clade to which it belongs. We emphasize that specimens submitted for cytogenetic analysis should be taxonomically identified. The non-identification of a specific sample triggers a series of problems, such as in the comparison with sister groups and eventual karyoevolutionary trajectories.

Our karyomorphometric approach was used primarily to reveal the chromosomal morphology of *M. iheringi*. Besides, future karyomorphometric comparisons among populations or even closely related lineages may serve as a basis for a possible delimitation of incipient lineages. For example, populations of *M. holmgreni* distributed on a North/South continuum of its distribution area diverged significantly in the length of their chromosomes, and the results were supported by flow cytometry analyses of the genome size (Cardoso et al. 2018b). Further, those populations were later identified to differ in the proportion of repetitive DNA by using FISH with microsatellite probes (Micolino et al. 2019a) Thus, the authors demonstrated the importance of using a standardized karyomorphometric approach coupled with genome size estimation to identify hidden chromosomal variations (see Cardoso et al. 2018b).

Finally, we used a FISH probe of the highly conserved TTAGG telomeric sequence in most insects (reviewed by Kuznetsova et al. 2020) to test the assumption that the putative inversion rearrangement occurred in *M. iheringi* and involved the telomere. However, we did not observe any signal for the probe at the interstitial telomeric sites, which would denote inversion involving the telomere. Indeed, the TTAGG sequence also seems to be fairly conserved in ants (Lorite et al. 2002), including fungus-farming ants such as *Acromyrmex striatus* Roger, 1863 (Pereira et al. 2018), *Mycetophylax* spp. (Micolino et al. 2019b), and *M. holmgreni* (Micolino et al. 2019a). In conclusion, we have described another ant species with the TTAGG sequence conserved in its telomeres, and we suggest a significant chromosomal mechanism, a major pericentric inversion, most likely occurred in *M. iheringi* and could have been involved in its diversification process.
